# 

**Published:** 1845-12

**Authors:** 


### CONTENTS

OF NO. VI.

### ORIGINAL COMMUNICATIONS.

ESSAYS AND CASES.

Art. I.—Remarks on the Diseases of Ohio, in a letter to the Editors. By John Dawson, M.D., of Jamestown, Ohio. 461
Art. II.—An Essay on the Mucous Membranes. Read before the Medical Society of Tennessee, in May, 1845. By J. Overton, M.D., of Nashville, Tenn. - - - - 471

REVIEWS.

Art. III.—Elements of Materia Medica and Therapeutics. By John P. Harrison, M.D., Professor of Materia Medica and Therapeutics in the Medical College of Ohio. Vol. II. Philadelphia: Thomas, Coperthwaite & Co. Cincinnati: Desilver & Burr. 1845. 8vo. pp. 619. - - 483
Art. IV—Animal Chemistry with reference to the Physiology and Pathology of Man. By Dr. J. Franz Simon, Fellow of the Society for the advancement of Physiological Chemistry at Berlin, &c., &c. Translated and edited by George E. Day, M.A. & L.M., Cantab., Licentiate of the Royal College of Physicians. - - - - - - 497

SELECTIONS FROM AMERICAN AND FOREIGN JOURNALS

On a Dangerous Form of Jaundice. - - - - - - - 509
On the Treatment of a peculiar form of Diarrhœa. - - - - 511
Forms of Disease in which Opiates are Indicated, - - - - 511
Treatment of Fistula in Ano by Ligature. - - - - - - 516
Observations on Relaxed Rectum. - - - - - - - 516
Poison by Tartaric Acid. - - - - - - - - - - 517

Contributions to the Practice of Midwifery. - - - - 518
On the Periodicity of Neuroses. - - - - - - - 522
Trousseau on the Indications of Anemia. - - - - - - 525
On the Causes and Prevention of Apoplexy and Paralysis. - - 527
Diseases of Women unconnected with Pregnancy and the Puerperal state. - - - - - - - - - 530
Violent Chorea St. Viti cured by Strychnine. - - - - 535
Tying the Subclavian Artery within the Scaleni Muscles of the left side for Aneurism. - - - - - - - 536
On a Simple Means of Arresting Bleeding from Leech Bites. - 537
Treatment to be persued in the Admission of Air into the Veins. 538
Effects of Ergot of Rye on the Parturient Female and her Offspring. 538

ORIGINAL INTELLIGENCE.

To the Physicians and Meteorological Observers of the Valley of the Mississippi and the Lakes. - - - - - 541
Gross's Pathological Anatomy. - - - - - - - - 542
Death of Dr. Houghton. - - - - - - - - - 543
Dickson's Essays on Pathology and Therapeutics. - - - 543
Strychnia in Paralysis, - - - - - - - - - 544
To our Correspondents, - - - - - - - - - 545
Alabama Medical University, - - - - - - - - 546
The Naturalist, - - - - - - - - - - 546
The New York Medical and Surgical Reporter, - - - 547
New York Medical Intelligencer, - - - - - - - 547
Stockton's Dental Intelligencer, - - - - - - - 548
Medical Institute of Louisville, - - - - - - - 548

